# SuperPolymyxin™ Medium for the Screening of Colistin-Resistant Gram-Negative Bacteria in Stool Samples

**DOI:** 10.3389/fmicb.2018.02809

**Published:** 2018-11-21

**Authors:** Sara M. Przybysz, Carlos Correa-Martinez, Robin Köck, Karsten Becker, Frieder Schaumburg

**Affiliations:** ^1^Institute of Medical Microbiology, University Hospital Münster, Münster, Germany; ^2^Institute of Hygiene, University Hospital Münster, Münster, Germany; ^3^Institute of Hygiene, DRK Kliniken Berlin, Berlin, Germany

**Keywords:** Colistin, Resistance, Screening, Stool, *mcr*-1

## Abstract

Colistin is one of the last resort antimicrobials for the treatment of infections caused by multidrug-resistant Gram-negative bacteria. After the emergence of transferable colistin resistance genes (*mcr*-1–5), a reliable culture-based screening method to detect colonization with colistin-resistant Gram-negative bacteria (CRGN) is needed. The objective of this study was to test the performance of SuperPolymyxin™ medium to screen for CRGN in stool samples and to compare different methods for the confirmation of colistin resistance (e.g., Etest®, broth microdilution [BMD], and the Rapid Polymyxin™ NP test). Colonization with CRGN was analyzed in a prospective cohort study among travelers. Stool samples (Fecal Transwab^TM^) taken before, during and after travel were cultured on SuperPolymyxin™ agar. Every phenotypically different colony was subcultured for species identification using MALDI-TOF mass spectrometry. Susceptibility to colistin was tested using Etest® and confirmed by BMD and the Rapid Polymyxin™ NP test. In total, 128 participants provided 1,495 stool samples. After culture on SuperPolymyxin™ medium (37°C, 24–48 h), 1,851 phenotypically different colonies were isolated. Isolates belonging to intrinsically colistin-resistant genera (e.g., *Morganella, Providencia, Proteus*) or *Stenotrophomonas maltophilia* were excluded from further analysis (*n* = 421). Among the remaining 1,430 isolates, colistin resistance was confirmed in 279 by Etest® (19.5%) and 218 by BMD (15.3%). The Rapid Polymyxin™ NP test was compared with BMD (reference) to detect colistin resistance (specificity: 88.6%, sensitivity 71.1%). SuperPolymyxin™ medium is suitable to screen for fecal colonization with CRGN. The high proportion of colistin-susceptible isolates growing on SuperPolymyxin™ medium caused a high workload. The confirmation of CRGN with the Rapid Polymyxin™ NP Test could be a less labor-intensive alternative to BMD.

## Introduction

The emergence and spread of antimicrobial resistance is currently one of the biggest challenges in health care worldwide. This is particularly true for Gram-negative bacteria. In the past decades, carbapenems were considered as safe and efficient antimicrobials for the treatment of infections with extended-spectrum β-lactamase (ESBL)-producing *Enterobacterales* or non-fermenting bacteria (e.g., *Pseudomonas aeruginosa, Acinetobacter baumannii*). Today, the increase and global spread of carbapenem-resistant bacteria raise serious concerns and physicians can find themselves “beamed back” to the pre-antimicrobial era as only very few compounds are available to treat infections with these multidrug-resistant microorganisms. For example, 13 of 38 European countries reported in 2015 an inter-regional dissemination or even an endemic occurrence of carbapenemase-producing *Enterobacterales* (Magiorakos et al., 2013; Albiger et al., 2015). In addition, carbapenemase-producing *Enterobacterales* were also detected in livestock, companion animals, seafood and wildlife (Köck et al., 2018).

Although parenteral colistin (Polymyxin E) was not considered safe (e.g., nephro- and neurotoxicity) and effective in the past, it experiences a revival in human medicine nowadays due to the lack of alternative antimicrobial agents to treat infections due to carbapenem-resistant bacteria. Moreover, colistin is widely used in veterinary medicine to treat diarrhea in poultry and pig production systems. In 2012, the European Union estimated that the use of polymyxins in food-producing animals was 600-times higher than in humans (Skov and Monnet, 2016).

Colistin is a decapeptide with a poor resorption after oral administration due to its hydrophilic properties. For the treatment of multidrug-resistant Gram-negative bacteria, it is given intravenously as the prodrug colistin methanesulfonate (colistimethate) (Grégoire et al., 2017). Colistin interacts with lipopolysaccharides on the outer membrane of Gram-negative bacteria and causes membrane damage leading to bacterial death (Grégoire et al., 2017). Some genera are intrinsically resistant to colistin (e.g., *Burkholderia, Hafnia, Morganella, Proteus, Providencia, Serratia*) (Leclercq et al., 2013; Jayol et al., 2017). However, acquired colistin resistance has been reported, which is either encoded on the bacterial chromosome (e.g., mutations in *lpxA, lpxC, lpxD, pmrA, pmrB, mgrB*), or on transferable plasmids (e.g., *mcr-1–5*) (Nordmann et al., 2016a; Grégoire et al., 2017). Recently, *Enterobacterales* harboring *mcr* genes have emerged in livestock (Irrgang et al., 2016; Kieffer et al., 2017; Wang et al., 2017). Hence, *mcr* genes (mainly *mcr-1*) are currently the main mechanism of colistin resistance in livestock farming and *mcr* positive colistin-resistant Gram-negative bacteria (CRGN) can now be found in humans, breeding animals (e.g., pigs, poultry) or even “filth flies”(Guenther et al., 2017; Wang et al., 2017; Onwugamba et al., 2018). International travel also contributes to the spread of colistin-resistant bacteria, particularly through tourists from South-East Asia (Arcilla et al., 2016).

Effective screening media are needed to detect individuals colonized with CRGN. Recently, a colistin resistance screening agar was developed by Nordmann et al. (SuperPolymyxin™ medium). To the best of our knowledge this is currently the only available selective agar for the screening of colistin-resistant bacteria (Nordmann et al., 2016a). SuperPolymyxin™ medium is based on eosin methylene blue, which is selective for Gram-negative bacteria. It also contains colistin, daptomycin and amphotericin B. Presumptive lactose-fermenting species grow in darkblue-brown colonies, while lactose-non-fermenters grow colorless or light lavender. In addition, the medium allows for the differentiation of lactose-fermenters as *Escherichia coli* colonies have a characteristic metallic green sheen and colonies of *Enterobacter* spp. and *Klebsiella* spp. are brown, dark-centered and mucoid. This SuperPolymyxin™ medium was already challenged with a selection of well-characterized colistin-susceptible and -resistant isolates (Nordmann et al., 2016a; Abdul Momin et al., 2017; Jayol et al., 2018).

Several test are available for the detection of colistin resistance but they are either labor intensive (broth microdilution [BMD]) or have a high rate of very major errors (Etest®) (Matuschek et al., 2018). Recently, the Rapid Polymyxin™ NP test was developed by Nordmann et al., which can detect colistin resistance within 2 h (Nordmann et al., 2016b). Briefly, the test is based on the metabolism of glucose by viable cells leading to a decrease of the pH-value. The change in pH is indicated by phenol-red. In case of colistin resistance, bacteria can grow in the presence of colistin (3.75 mg/L) and the test suspension turns from red to yellow (Nordmann et al., 2016b). This Rapid Polymyxin™ NP test could be a suitable alternative to BMD or Etest®.

The objective of this study was now to assess the test performance and applicability of SuperPolymyxin™ medium in routine screening of fecal samples and to compare different methods for the confirmation of colistin resistance (e.g. Etest®, BMD, and the Rapid Polymyxin™ NP test).

## Materials and methods

### Ethics

Ethical clearance was obtained from the ethical committee of the medical faculty of “Westfälische Wilhelms-Universität,” Münster, Germany (approval number: 2014-013-f-S). All participants signed a written informed consent prior to any study-related procedures.

### Fecal samples and media characteristics

Fecal samples were self-collected by international travelers who took part in a prospective cohort study on the import of antimicrobial-resistant bacteria from abroad (Münster, Germany, April 2016–April 2018). Participants were trained to collect samples strictly avoiding cross-contamination from the environment by either sticking the whole swab in the stool or by swabbing the stool from the anal region before cleaning. The participants provided stool samples in Cary-Blair medium (Fecal Transwab™, MWE medical wire, Corsham, England) once before, during and after the trip (Arcilla et al., 2017). The fecal swabs were stored at room temperature until being cultured. For that purpose, 10 μl of the inoculated Cary-Blair medium were streaked on SuperPolymyxin™ medium (three-phase streak technique) and cultured for 24–48 h at 37°C under aerobic conditions (Nordmann et al., 2016a).

### Bacterial cultures

**Figure 1:**
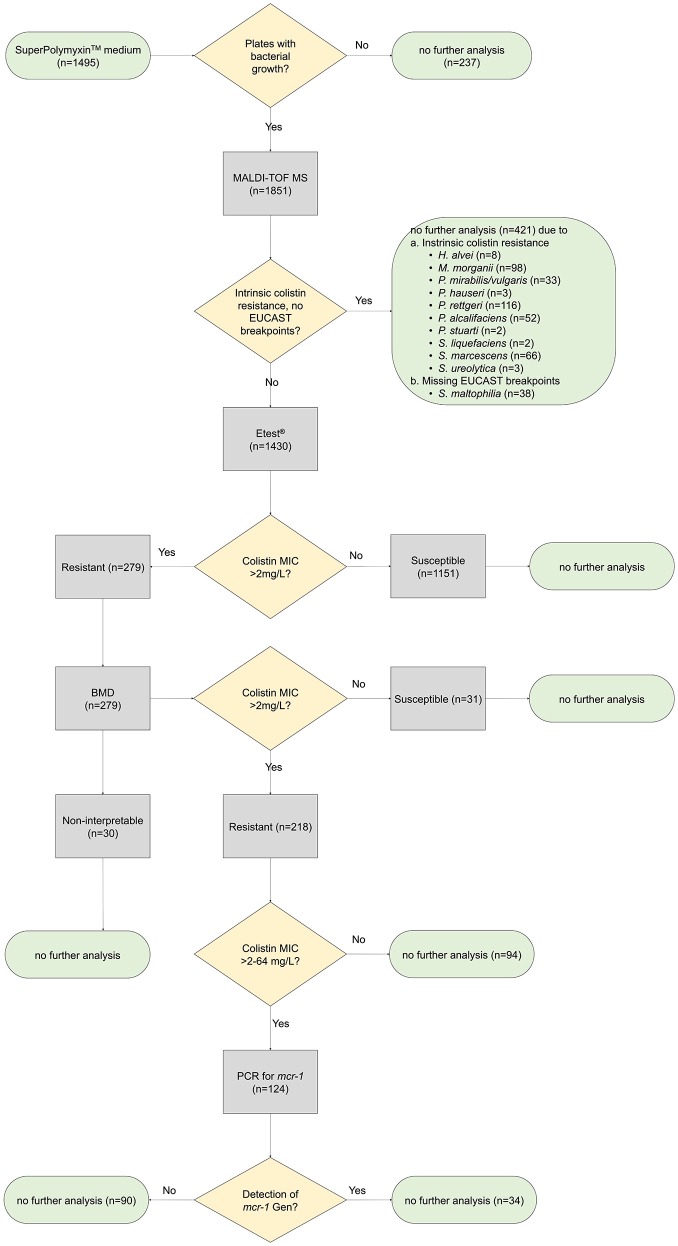
Study procedure. Minimum inhibitory concentrations (MIC) of all colistin non-intrinsically resistant bacterial species growing on SuperPolymyxin™ medium were tested by gradient diffusion test (Etest®, bioMérieux) and broth microdilution (BMD) to confirm colistin resistance. Presence of the transferable resistance determinant *mcr-1* was tested by PCR.

One of each phenotypically different colony growing on SuperPolymyxin™ medium was subcultured on Columbia blood agar for species identification using matrix-assisted laser desorption/ionization time-of-flight (MALDI-TOF) mass spectrometry (Bruker, Bremen, Germany) applying the database MBT Compass 4.1. All intrinsically resistant species were removed from further susceptibility testing (e.g., *Hafnia alvei, Morganella morganii, Proteus hauseri, Proteus vulgaris, Proteus mirabilis, Providencia rettgeri, Providencia stuarti, Providencia alcalifaciens, Serratia liquefaciens, Serratia marcescens, Serratia ureolytica*) (Magiorakos et al., 2012; Leclercq et al., 2013; Jayol et al., 2017; Saly et al., 2017) (Figure 1)*. Stenotrophomonas maltophilia* was excluded due to missing EUCAST breakpoints for colistin.

### Susceptibility testing

All isolates being not intrinsically resistant to colistin were further tested using colistin Etest® (bioMérieux, Marcy l'Étoile, France) on Mueller-Hinton agar (BD Diagnostics, Heidelberg, Germany) according to the manufacturer's instruction. Minimum inhibitory concentrations (MIC) were interpreted according to EUCAST clinical breakpoints (Version 8.0; colistin-susceptible: MIC ≤ 2 mg/L, colistin-resistant: MIC >2 mg/L). MICs from Etest® were rounded to the next doubling step for comparison with MIC from broth microdilution (Schaumburg et al., 2017).

Colistin resistance was confirmed by the BMD reference method (Sensititre^TM^ FRCOL, Thermo Fisher Scientific, Wesel, Germany) according to the manufacturer's instruction. Briefly, bacterial suspensions (0.5 McFarland) were prepared in Mueller-Hinton broth with TES buffer and transferred to the microtitre plates (range of colistin concentration: 0.12–128 mg/L). The results were read after 18–24 h at 35°C. Test results with “skipped wells” (i.e., no growth in one well but growth in a well with higher colistin concentration) were considered non-interpretable (Poirel et al., 2017).

The Rapid Polymyxin™ NP Test was assessed as a rapid and cheap alternative to BMD to confirm colistin resistance (Nordmann et al., 2016b). The test was performed in triplicate. In case of inconsistent results, the final interpretation of the test was based on the result as suggested by the majority of the three test runs.

*E. coli* NCTC 13846 (DSMZ 105182, colistin-resistant) and *P. aeruginosa* ATCC 27853 (colistin-susceptible) were used as controls.

#### mcr-1 screening

All isolates having a MIC >2–64 mg/L as tested by BMD were screened for the presence of *mcr-1* by isothermal amplification (eazyplex® SuperBug *mcr-1*, AmplexDiagnostics GmbH, Gars-Bahnhof, Germany). This range was chosen as almost all *mcr* positive CRGN showed a colistin MIC ≤ 64 mg/L (Nordmann et al., 2016a; Eiamphungporn et al., 2018; Poirel et al., 2018).

### Statistics

Statistical analyses were performed with “R” (package “epiDisplay” and “caret”). The 95% confidence interval (95% CI) of error rates (major and very major errors) and the categorical agreement between BMD and Etest® or Rapid Polymyxin™ NP Test was calculated with Wilson procedure without a correction for continuity.

## Results

In total, 128 participants were included who provided 1,495 fecal swabs (median number of swabs per participant: 12, range: 2–23). Of these, 1,258 (84.2%) showed growth on SuperPolymyxin™ medium. No growth was detected on 237 plates (15.8%, Figure 1).

The median number of phenotypically different colonies growing on SuperPolymyxin™ medium was one colony (range: 1–3). In total, 1,851 phenotypically different colonies from SuperPolymyxin™ medium were subcultured for species identification (MALDI-TOF MS). We identified 47 different species.

In total, 421 isolates (22.7%) belonging to the following species were considered intrinsically colistin resistant and were excluded from further testing: *P. rettgeri* (*n* = 116), *M. morganii* (*n* = 98), *S. marcescens* (*n* = 66), *P. alcalifaciens* (*n* = 52), *P. mirabilis/vulgaris* (*n* = 33), *H. alvei* (*n* = 8)*, P. hauseri* (*n* = 3), *S. ureolytica* (*n* = 3), *P. stuarti* (*n* = 2) and *S. liquefaciens* (*n* = 2, Figure 1). *S. maltophilia* (*n* = 38) was excluded due to missing EUCAST breakpoints.

Overall, 77.3% of the isolates from SuperPolymyxin™ agar (*n* = 1,430) were Gram-negative species that are normally susceptible to colistin, such as *Klebsiella pneumoniae* (*n* = 363), *E. coli* (*n* = 251), *Enterobacter cloacae* (*n* = 188), *Klebsiella variicola* (*n* = 168), *Enterobacter asburiae* (*n* = 147), *Pseudomonas aeruginosa* (*n* = 72), *Enterobacter kobei* (*n* = 46), *Raoultella ornithinolytica* (*n* = 39), *Enterobacter aerogenes* (*n* = 38), *Citrobacter freundii* (*n* = 34), *Comamonas testosteroni* (*n* = 16) and others (*n* = 68). Only one Gram-positive species (*Enterococcus* sp.) grew on the medium.

The non-intrinsically resistant species (*n* = 1,430) were screened for colistin resistance by Etest®. Colistin resistance was detected in 19.5% (279/1,430) of the isolates. Although Etest® is a convenient method to measure MICs, it is not recommended for colistin susceptibility testing due to false susceptible results (Matuschek et al., 2018). We confirmed colistin resistance with BMD in all isolates being tested resistant by Etest® (*n* = 279). BMD confirmed colistin resistance in 78.1% (218/279). Thus, 15.3% (218/1,430) of all non-intrinsically colistin resistant species growing on SuperPolymyxin™ medium were colistin resistant.

To assess the proportion of *mcr-1* mediated colistin resistance, all isolates exhibiting a colistin MIC between >2–64 mg/L (*n* = 124) were screened for *mcr-1* by PCR. This comprised *Acinetobacter junii* (*n* = 1), *Delftia acidovorans* (*n* = 1), *E. coli* (*n* = 39), *E. cloacae* (*n* = 39), *E. asburiae* (*n* = 17), *R. ornithinolytica* (*n* = 5), *E. kobei* (*n* = 13), *C. testosteroni* (*n* = 6), *Ochrobactrum* sp. (*n* = 1), *Pseudomonas protegens* (*n* = 1), and *Yokenella regensburgei* (*n* = 1). A total of 26.6% (*n* = 34) were *mcr-1* positive (33 *E. coli*, 1 *E. asburiae*).

Since Etest® can underreport colistin resistance, we tested the Etest® performance in our setting using BMD as reference. For that purpose, we selected 130 consecutively collected species from our samples. Twelve isolates were excluded due to non-interpretable results in BMD (skipped wells, Table 1).

**Table 1:** Comparison of Etest® and broth microdilution for colistin susceptibility testing.

		**Broth microdilution (*****n*****)**	
		**Resistant**	**Susceptible**	**Total**
Etest® (*n*)	Resistant	37	3	40
	Susceptible	9	69	78
	Total	46	72	118^a^

a *12 isolates were excluded due to non-interpretable results in BMD (skipped wells)*.

The essential agreement between Etest® and BMD was 59.3% (70/118, 95%CI: 50.3%−67.7%) and the category agreement was 89.8% (106/118, 95%CI: 83.1–94.1%). Wrong Etest® results were either due to major errors (false resistant: 3/118, 2.5%, 95%CI: 0.9–7.2%) or very major errors (false susceptible: 9/118, 7.6%, 95%CI: 4.1–13.9%).

The specificity and sensitivity of Etest® (reference: BMD) was 95.8% and 80.4%, respectively. The negative and positive predictive values for the detection of colistin resistance using Etest® were 88.5 and 92.5%, respectively (accuracy: 89.8%).

BMD is recommended for colistin susceptibility testing, but it is labor intensive and time consuming. Therefore, we tested, if the Rapid Polymyxin™ NP Test can be applied to rapidly confirm or rule out colistin resistance. For that purpose, the same set of consecutively selected isolates (*n* = 130) was tested with Rapid Polymyxin™ NP Test (merged test results of triplicate runs) and BMD (reference).

Test results of the Rapid Polymyxin™ NP Test were not evaluable in three subsequent test runs for *P. alcalifaciens* (*n* = 4), *C. testosteroni* (*n* = 2), *Acinetobacter junii* (*n* = 1), *Pseudomonas fluorescens* (*n* = 1), *E. kobei* and *P. aeruginosa* (*n* = 1) due to no growth in the growth control. These isolates were excluded for the calculation of the test performance. Additionally, 12 isolates were excluded due to skipped wells in BMD.

The category agreement between Rapid Polymyxin™ NP Test and BMD was 82.1% (89/108, 95%CI: 74.1–88.4%). Disagreement was either due to major errors (false resistant: 8/108, 7.4%, 95%CI: 3.8–13.9%) or very major errors (false susceptible: 11/108, 10.2%, 95%CI: 5.8–17.3%).

The specificity and sensitivity of Rapid Polymyxin™ NP Test compared to BMD were 88.6 and 71.1%, respectively. The negative and positive predictive values to detect colistin resistance using Rapid Polymyxin NP Test were 84.9 and 77.1%, respectively (accuracy: 91%).

The test performance was impaired, if only the first test run of Rapid Polymyxin™ NP Test and not the merged results of the triplicate testing were considered (specificity: 85.7%, sensitivity: 63.4%, negative predictive value: 80.0%, positive predictive value: 72.2%).

## Discussion

We tested the applicability of SuperPolymyxin™ medium and found that it is suitable to detect colistin resistance (including *mcr-1* positive isolates) in human fecal samples but it was associated with high workload.

The high proportion of positive SuperPolymyxin™ medium (growth after 48 h, 84.2%) in our study is in contrast to a recent report on hospitalized patients where bacterial growth was found on 17/41 agars (41.5%) after inoculation from rectal swabs (Jayol et al., 2018). In our study, the participants stored the samples at room temperature during their travel and bacteria might have multiplied during this time. The different rates of growth on SuperPolymyxin™ medium (84.2 vs. 41.5%) might be due to an inoculum effect as colistin-susceptible isolates were shown to grow on SuperPolymyxin™ medium at ≥10^6^ CFU/mL (Nordmann et al., 2016a). If this played a role in our study, one should expect a higher proportion of colistin susceptible bacteria in early compared to late samples due to longer storage times. However, the proportion of susceptible isolates (according to Etest®) was similar throughout the study fluctuating between 16 and 27% (data not shown).

In total, 279 of 1,430 isolates were found to be colistin-resistant by Etest®, which was confirmed in 218 isolates (78.1%) by BMD. This is in line with a recent comparison of different Etest® manufacturers (reference: BMD) where the categorical agreement was 79–85% (Matuschek et al., 2018).

To detect the 218 isolates with acquired colistin resistance, we had to perform 1,851 MALDI-TOF MS tests (to rule out intrinsically resistant isolates) and 1,430 Etest® cultures, thus experiencing a relevant workload. Most likely, this might be due to an inoculum effect due to bacterial growth during storage at room temperature (Nordmann et al., 2016a). A low quality of in-house SuperPolymyxin™ medium is unlikely since we strictly followed the recommendations (e.g., 7 days shelf life, storage of colistin stock solution in glass tubes) (Nordmann et al., 2016a). Others recently showed that SuperPolymyxin™ medium could even be used for up to 4 weeks (Abdul Momin et al., 2017).

Several studies evaluating SuperPolymyxin™ medium did not use clinical samples but monocultures or defined mixed cultures of different species (n: 2–9) and therefore cannot be compared with our study as we used fecal samples (Nordmann et al., 2016a; Abdul Momin et al., 2017). Other reports analyzed rectal colonization rates with *mcr-1* positive isolates in pigs (98%) and hospitalized patients (0%) using SuperPolymyxin™ medium (Kieffer et al., 2017; Saly et al., 2017; Jayol et al., 2018). These studies did not focus in detail on the proportion of colistin-susceptible isolates growing on SuperPolymyxin™ medium. Notably the high number of colistin-susceptible isolates growing on SuperPolymyxin™ medium caused the major workload (Figure 1).

In a subset of 130 consecutively collected isolates, we studied the test performance of Etest® (reference: BMD) in our setting. Here, the categorical agreement (89.9%) was even slightly better compared to a recent study with 75 isolates (79–85%) depending on the manufacturer of the gradient diffusion test (Table 1) (Matuschek et al., 2018). Since the very major error rate (i.e., false susceptible isolates) of Etest® in our setting was 7.6%, we might have missed approximately 8% of non-intrinsically colistin-resistant isolates using our approach (i.e., screening for colistin resistance with Etest® and confirming with BMD, Figure 1). We decided to use Etest® as a screening method and BMD for confirmation as BMD was considered to cause an inapplicable workload due to the high number of isolates. However, an alternative to confirm/rule-out colistin resistance is the Rapid Polymyxin™ NP Test. (Table 2). However, Compared to Etest®, Rapid Polymyxin™ NP Test had a lower specificity (95.8 vs. 88.6%) and sensitivity (80.4 vs. 71.1%) to detect colistin resistance (Tables 1, 2).

**Table 2:** Comparison of the Rapid Polymyxin™ NP Test and broth microdilution to detect colistin resistance.

		**Broth microdilution (*****n*****)**	
		**Resistant**	**Susceptible**	**Total**
Rapid Polymyxin™ NP Test (*n*)	Resistant	27	8	35
	Susceptible	11	62	73
	Total	38	70	108^a^

a *10 isolates were excluded the due to non-evaluable results in Rapid Polymyxin™ NP Test (no growth in the growth control) and 12 isolates were excluded due to non-interpretable results in broth microdilution (skipped wells)*.

Although our study provides valuable data for the screening of colistin resistance in fecal samples, some limitations need to be addressed. First, we did not apply broth microdilution to confirm colistin resistance in all isolates growing on SuperPolymyxin™ medium but only used BMD for confirmation of colistin-resistant isolates according to Etest®. Since Etest® can have high false-susceptible rates, we might have missed additional colistin-resistant isolates. Second, we did not screen for newer *mcr*-members (i.e., *mcr-2*–*mcr-5*). These members are still very rare and the European Center for Disease Prevention and Control currently recommend only a screening for *mcr-1* (European Centre for Disease Prevention Control, 2016; Sun et al., 2018). Third, since we only tested fecal samples from international travelers, we are unable to draw any conclusions on the test performance of SuperPolymyxin medium^TM^ when screening hospitalized patients, animals or environmental samples for colistin-resistant isolates.

In conclusion, SuperPolymyxin™ medium is suitable to screen for fecal colonization with colistin-resistant isolates but is associated with a high workload due to a high proportion of colistin-susceptible isolates growing on SuperPolymyxin™ medium. The confirmation of colistin resistance with the Rapid Polymyxin™ NP Test is a cheap and but less-accurate alternative to BMD.

## Author contributions

KB, RK, SP, and FS designed the study. SP, CC-M, and FS performed sampling and microbiological analyses. SP and FS analyzed the data. SP and FS drafted the manuscript. All authors reviewed and agreed on the final version of the manuscript. All authors have commented and agreed on the final version of the manuscript.

### Conflict of interest statement

The authors declare that the research was conducted in the absence of any commercial or financial relationships that could be construed as a potential conflict of interest.
